# Collinearity in ecological niche modeling: Confusions and challenges

**DOI:** 10.1002/ece3.5555

**Published:** 2019-08-20

**Authors:** Xiao Feng, Daniel S. Park, Ye Liang, Ranjit Pandey, Monica Papeş

**Affiliations:** ^1^ Institute of the Environment University of Arizona Tucson AZ USA; ^2^ School of Natural Resources and the Environment University of Arizona Tucson AZ USA; ^3^ Department of Organismic and Evolutionary Biology Harvard University Cambridge MA USA; ^4^ Department of Statistics Oklahoma State University Stillwater OK USA; ^5^ Department of Integrative Biology Oklahoma State University Stillwater OK USA; ^6^ Department of Ecology and Evolutionary Biology University of Tennessee Knoxville TN USA

**Keywords:** bioclim, collinearity shift, ecological niche, mammal, model transfer, predictor selection, species distribution model

## Abstract

Ecological niche models are widely used in ecology and biogeography. Maxent is one of the most frequently used niche modeling tools, and many studies have aimed to optimize its performance. However, scholars have conflicting views on the treatment of predictor collinearity in Maxent modeling. Despite this lack of consensus, quantitative examinations of the effects of collinearity on Maxent modeling, especially in model transfer scenarios, are lacking. To address this knowledge gap, here we quantify the effects of collinearity under different scenarios of Maxent model training and projection. We separately examine the effects of predictor collinearity, collinearity shifts between training and testing data, and environmental novelty on model performance. We demonstrate that excluding highly correlated predictor variables does not significantly influence model performance. However, we find that collinearity shift and environmental novelty have significant negative effects on the performance of model transfer. We thus conclude that (a) Maxent is robust to predictor collinearity in model training; (b) the strategy of excluding highly correlated variables has little impact because Maxent accounts for redundant variables; and (c) collinearity shift and environmental novelty can negatively affect Maxent model transferability. We therefore recommend to quantify and report collinearity shift and environmental novelty to better infer model accuracy when models are spatially and/or temporally transferred.

## Graphical abstract

Excluding highly correlated variables does not affect Maxent model performance. Model transfer may lead to novel environment and collinearity shift, while both can negatively affect model performance.

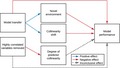

## 1 INTRODUCTION

Species are distributed nonrandomly across geographic space. The relationship between a species' distribution and environmental conditions is known as species' ecological niche (Grinnell, 1917; Hutchinson, 1957, 1978; Soberón, 2007), and the niche concept has received much attention and development (Chase & Leibold, 2003; James, Johnston, Wamer, Niemi, & Boecklen, 1984; Sax, Early, & Bellemare, 2013; Soberón & Nakamura, 2009). With the advancement of GIS techniques and rapid digitization and mobilization of museum specimens, ecological niche modeling (ENM), also referred as species distribution modeling (SDM), is increasingly used to quantify such relationships between species’ presences and environmental conditions (Peterson et al., 2011). Of the many ENM algorithms developed, the most widely used, by far, is Maxent (Phillips, Dudík, & Schapire, 2004). Maxent requires only presence data as input and estimates species’ relative occurrence rates (Yackulic et al., 2013) by minimizing the relative entropy between the probability densities of species’ presences and the training background (Elith et al., 2011). Maxent also provides a user‐friendly interface (Elith et al., 2006; Phillips & Dudík, 2008), and the publications introducing the Maxent algorithm to ecologists have been cited collectively tens of thousands of times (Joppa et al., 2013).

Maxent has been applied to a wide range of studies, including those related to discovering rare species (Fois, Fenu, Lombrana, Cogoni, & Bacchetta, 2015; Jackson & Robertson, 2011; Menon, Choudhury, Khan, & Peterson, 2010), conservation and invasive species management (Feng, Lin, Qiao, & Ji, 2015; Ficetola, Thuiller, & Miaud, 2007; Park & Potter, 2015a, 2015b; Roura‐Pascual, Brotons, Peterson, & Thuiller, 2009), and disease transmission (Escobar et al., 2015; Gonzalez et al., 2011). Concurrently, many methodological studies have aimed to optimize model performance. Studies have explored the effect of presence sample size (Hernandez, Graham, Master, & Albert, 2006; Jiménez‐Valverde, Lobo, & Hortal, 2009), spatial and/or environmental occurrence bias (Boria, Olson, Goodman, & Anderson, 2014; Park & Davis, 2017; Varela, Anderson, García‐Valdés, & Fernández‐González, 2014), various procedures of selecting pseudo‐absences (Barbet‐Massin, Jiguet, Albert, & Thuiller, 2012; Iturbide et al., 2015; Phillips et al., 2009), and designing a model training area that is ecologically valid (Anderson & Raza, 2010; Saupe et al., 2012). Additionally, studies have explored the selection of predictor variables using statistical approaches (correlation analysis, jackknifing, or contribution to model fit), as well as using knowledge about the species' ecology (Bucklin et al., 2015; Pliscoff, Luebert, Hilger, & Guisan, 2014; Synes & Osborne, 2011; Zeng, Low, & Yeo, 2016). Principal component analysis has also been used to reduce the dimensionality of the environmental dataset (De Marco Júnior & Nóbrega, 2018).

However, a lack of consensus still exists regarding whether and how predictor collinearity (i.e., the linear dependence among environmental predictor variables) should be treated in Maxent modeling. Indeed, we examined recent papers citing the major Maxent references (Phillips, Anderson, & Schapire, 2006; Phillips & Dudík, 2008; Phillips et al., 2004) and found that ~80% of papers never mentioned “collinearity” or “variable correlation” (Google Scholar accessed 6 November 2017; see Appendix S1; Table S1). The impacts of predictor collinearity are well documented in classical linear regression models (e.g., ordinary least square estimation in linear regression models). For example, if two variables are highly correlated, it becomes difficult to separate the individual effects of each variable. Also, models trained with correlated variables are prone to error when the correlation between variables changes in model transfer scenarios (Dormann et al., 2013; Meloun, Militký, Hill, & Brereton, 2002). Ideally, one would consider biologically meaningful variables over the issue of collinearity (Dormann et al., 2013; Tanner, Papeş, Elmore, Fuhlendorf, & Davis, 2017). However, the problem of collinearity is difficult to avoid in the process of selecting biologically meaningful variables as many commonly applied environmental predictors are highly correlated and/or nonindependent (Jiménez‐Valverde, Nakazawa, Lira‐Noriega, & Peterson, 2009). In practice, the rule‐of‐thumb method in dealing with collinearity is to minimize its potential effect by selecting variables whose correlation coefficients are below a certain threshold (e.g., |*r*| <0.7 in Dormann et al. (2013) or <0.4 in Suzuki, Olson, & Reilly (2008)). However, rules established for classical regression models may not directly apply to Maxent modeling, and there are two competing views regarding the issue of collinearity in Maxent. Some have argued that, because Maxent can regulate model complexity by downplaying the importance of redundant variables, the algorithm is robust to issues of collinearity (Elith et al., 2011; Phillips & Dudík, 2008; Shcheglovitova & Anderson, 2013). Others attest that Maxent may partially handle collinearity, but predictor collinearity should be minimized by the user (Merow, Smith, & Silander, 2013). Though both views are well represented in the ENM literature, to our knowledge, to date there have been no empirical examinations of the effects of predictor collinearity on Maxent models.

The influence of collinearity on regression‐type models can be twofold: (a) the effect on model training caused by the degree of predictor collinearity and (b) the effect on model transfer caused by differences in the correlation structure of predictor variables between training and testing (or projecting) regions (i.e., collinearity shift). Thus, the issue of collinearity in Maxent must be considered from the perspective of model transfer, that is, transferring a model across space and/or time to different environmental conditions (Elith & Leathwick, 2009; Guisan & Thuiller, 2005; Peterson et al., 2011). When models are not transferred, collinearity effects will likely depend on the mechanism that impacts the model training per se; in the case of model transfer, collinearity shift may become the dominating mechanism. In the context of model transfer, another factor that influences model performance is model extrapolation, that is, the ability to make predictions in environmental conditions beyond those used in model training (Gelman & Hill, 2007). Previous studies have shown that environmental novelty is negatively associated with model performance (Fitzpatrick et al., 2018; Owens et al., 2013; Qiao et al., 2019). Therefore, environmental novelty should be considered together with collinearity shift in a model transfer scenario.

Here, we aim to clarify the effects of collinearity on Maxent models, especially in the context of model transfer. Specifically, our objectives are to (a) determine whether the performance of Maxent models declines in model transfer scenario compared with nontransfer scenario and determine whether the commonly adopted variable selection strategy (i.e., remove highly correlated variables) is effective in improving Maxent model performance, (b) assess the effect of variable selection strategy in controlling the degree of predictor collinearity and assess the effect of model transfer on environmental novelty and collinearity shift, and (c) determine the effect of environmental novelty, degree of predictor collinearity, and collinearity shift on model transfer performance. To achieve our objectives, we simulated scenarios of model transfer and nontransfer, selected predictors with and without considering collinearity, and quantified model performance, degree of predictor collinearity in the training region, and environmental novelty and collinearity shift between training and testing regions.

## 2 MATERIAL AND METHODS

**Figure 1:**
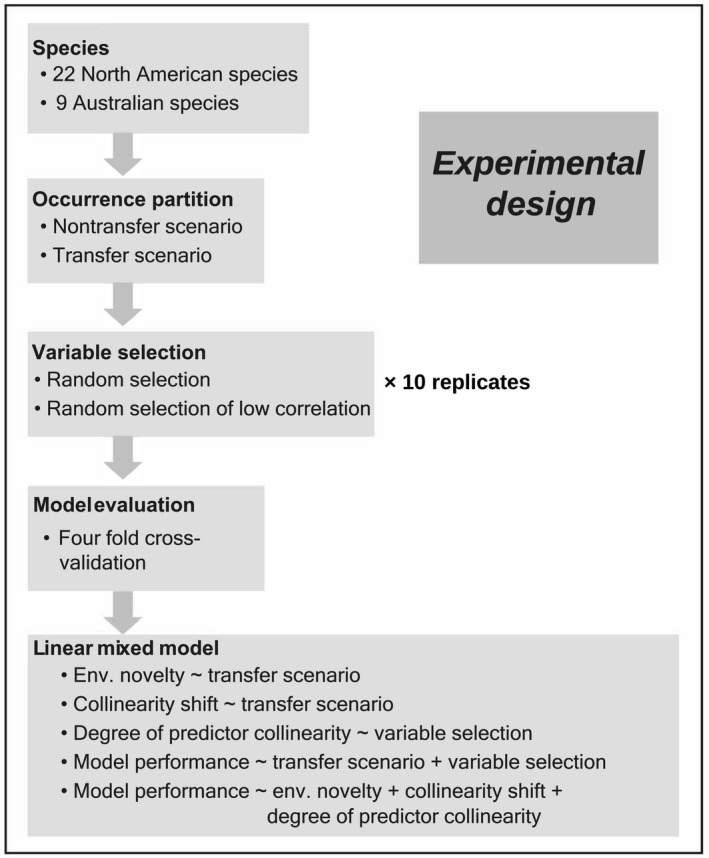
Overview of experimental design

To address our first objective, determining whether model transfer and variable selection strategy influence Maxent performance, we compared model performance between model transfer and nontransfer scenarios and between two variable selection strategies that either included or excluded highly correlated predictors (Figure 1). To address the second objective, assessing the effect of variable selection strategy in controlling the degree of predictor collinearity and assessing the effect of model transfer on environmental novelty and collinearity shift, we quantified the degree of predictor collinearity in the training region and quantified environmental novelty and collinearity shift between training and testing regions, and compared them under different variable selection strategies and model transfer scenarios (Figure 1). To address the third objective, we analyzed the relationship between model performance and environmental novelty, degree of predictor collinearity, and collinearity shift (Figure 1).

### 2.1 Study system

To conduct our ENM experiments, we focused on two groups of mammal species from North America and Australia that have distinct and well‐sampled distributions. We examined range maps from the International Union for Conservation of Nature (IUCN; http://www.iucnredlist.org/) of all North American carnivore mammals listed in Kays and Wilson (2009) and selected only species that are either endemic to, or have the majority of their distributional area in, North America. We downloaded occurrence records from the Global Biodiversity Information Facility (http://www.gbif.org/; accessed on 19 August 2016) and only retained records associated with verifiable sources (i.e., specimens and publications), to limit the inclusion of erroneous records. To avoid any marginal occurrences that may bias models (Soley‐Guardia et al., 2016), we removed occurrences outside the IUCN range maps of each species. To reduce the spatial aggregation of occurrences, we adopted a systematic sampling approach (Fourcade, Engler, Rödder, & Secondi, 2014) [similar as the spatial filtering approach (Araújo, 2006; Boria et al., 2014)] by which only one occurrence is kept within a spatial window. A broad spatial window may be effective in reducing spatial bias but may also eliminate information that holds ecological values (Fourcade et al., 2014), and vice versa for a small window. We used a spatial window of 30 arc‐minutes (~55 km at equator), in between those used in previous studies [2° and 12 arc‐minutes in Fourcade et al. (2014); 10 km or approximately 5.5 arc‐minutes in Boria et al. (2014)].To ensure a baseline of model performance, we further excluded species with low numbers of unique occurrences (smaller than 15; Papeş & Gaubert, 2007). Our final dataset comprised 22 carnivorous mammal species (Table S2). Using the criteria outlined above, we also selected nine marsupial species in Australia (Menkhorst & Knight, 2010).

We used 19 climatic variables at 2.5 arc‐minute resolution from the WorldClim dataset (version 1.4; Hijmans, Cameron, Parra, Jones, & Jarvis, 2005) as our pool of predictors, since climate has been shown to strongly influence the distribution of species (Parmesan, 2006; Walther et al., 2002). Furthermore, the WorldClim dataset is likely the most widely used climatic dataset in ENM; thus, this dataset allows us to replicate common practice. Indeed, this dataset has been cited over 10,000 times to date (Google Scholar; accessed 6 November 2017). We did not aim to select variables that have known mechanistic relationships with species’ distributions because such information is often unavailable or incomplete and commonly assumed rather than robustly established (Braunisch et al., 2013; Peterson et al., 2011). As our study addresses the effect of collinearity from a methodology perspective, we aimed to mimic typical practices in ENM literature.

### 2.2 Data partitioning and model transfer scenarios

**Figure 2:**
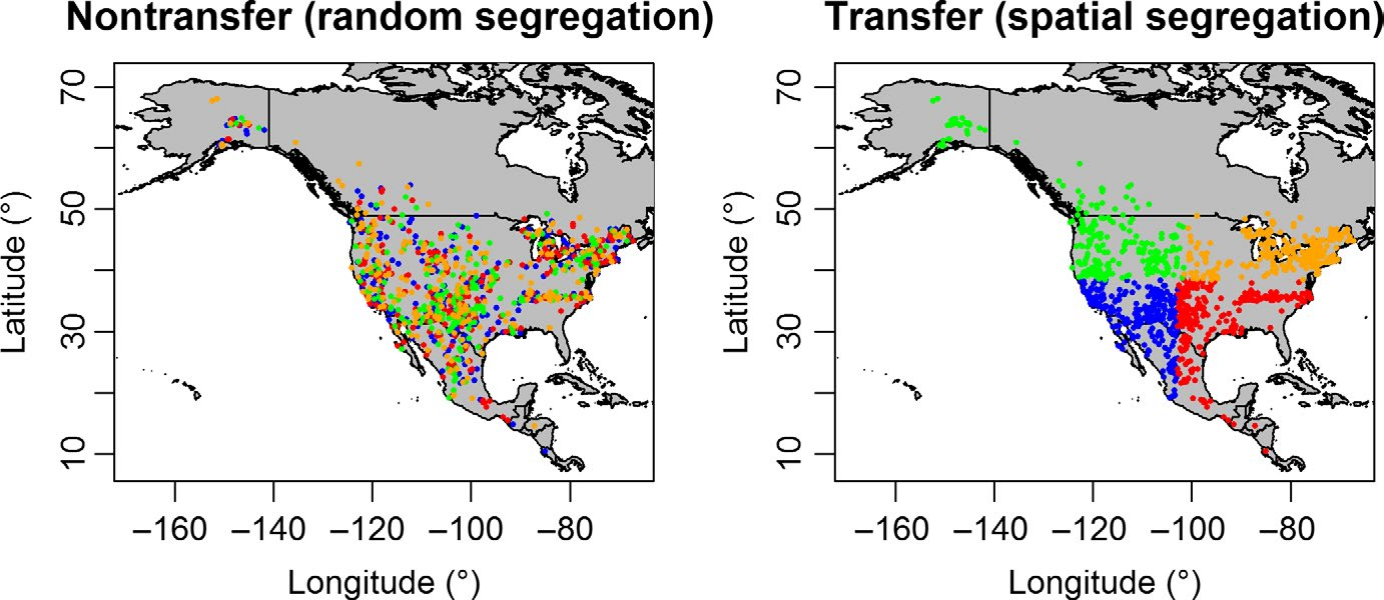
Occurrence data partition schemes for model transfer and nontransfer scenarios. The occurrences of each species (e.g., *Canis latrans* in North America, as shown in the figure) are separated into four groups (to be used in model training and testing), either randomly (nontransfer scenario; left panel) or spatially (transfer scenario; right panel). The four colors represent the four occurrence partitions

We partitioned the occurrence data for each species in two ways to simulate model transfer and nontransfer scenarios. We used the “checkerboard2” method (using two as aggregation factor; Muscarella et al., 2014) to simulate scenarios where models are not spatially transferred; this method is an advanced random segregation approach that decreases the effect of sampling bias (Hijmans, 2012). To simulate scenarios of model transfer across space, we used the “block” approach to partition our occurrence data spatially (Muscarella et al., 2014). For each species, we separated the occurrence dataset in geographic space into four sets using either the “checkerboard2” or “block” method, and used three sets for model training and one set for testing (Figure 2). These spatially segregated sets of occurrences (“block” partitions) are expected to vary in their climate compared with those based on the “checkerboard2” method (Muscarella et al., 2014).

We built two‐decimal degree buffers (approximately 220 km at equator) around each species occurrence point and used them as training regions (Anderson & Raza, 2010). We randomly selected 10,000 background points for Maxent from within the training regions. We used the default Maxent parameters (version 3.3.3k), including default feature and regularization settings, which were determined by an empirical study (Phillips & Dudík, 2008). It is possible that by tweaking the features and regularization parameters (Cobos, Peterson, Barve, & Osorio‐Olvera, 2019; Muscarella et al., 2014), different modeling settings could all achieve best performances, and some of the best performances metrics may be equally good (Feng, Anacleto, & Papeş, 2016). However, optimizing the model fully [e.g., in ENMeval (Muscarella et al., 2014) or kuenm (Cobos et al., 2019)] was not the goal of this study and the manipulated parameters are not known to directly influence how collinearity or environmental novelty is handled. In addition, the automatically determined features will be consistent among modeling replicates of the same species and thus will not affect subsequent comparisons of model performance. We used the same background points in model evaluation (see Muscarella et al., 2014). To evaluate model performance, we used one threshold‐independent evaluation index (i.e., area under the receiver operating characteristic curve; AUC) and two threshold‐dependent evaluation indices (i.e., true skill statistic (TSS) and sensitivity with a 5% omission rate threshold). AUC values can range from 0 to 1, with values above 0.5 indicating models better than random (Swets, 1979); TSS values can range from −1 to 1, with values above 0 indicating models better than random (Allouche, Tsoar, & Kadmon, 2006). Given that background points instead of absence data were used, omission error (false‐negative rate) is expected to have higher importance than commission error (false positive rate), so sensitivity (proportion of known presences predicted present; 0–1 values) was included as another evaluation index (Leroy et al., 2018; Lobo, Jiménez‐Valverde, & Real, 2008; Peterson, Papeş, & Soberón, 2008). Using this framework, we compared model performance in transfer versus nontransfer scenarios in downstream analyses (see Section 2.4). We conducted these analyses using the *raster* (Hijmans & van Etten, 2016) and *dismo* (Hijmans, Phillips, Leathwick, & Elith, 2017) packages in *R* (R Development Core Team, 2017).

### 2.3 Manipulating the degree of predictor collinearity

To determine the influence of collinearity on model performance, we used two variable selection strategies: random selection (V_Random_) and random selection of less correlated variables (V_RandomLowCor_). For V_Random_, we randomly selected a subset of predictors from a pool of environmental variables; V_Random_ serves as the control group for V_RandomLowCor_, as well as to represent studies that do not account for collinearity. For V_RandomLowCor_, we randomly selected a subset of predictors that are less correlated (based on random background points) using the 0.7 threshold for correlation coefficient (Dormann et al., 2013).

We repeated both methods of variable selection 10 times for each species to account for stochasticity in variable selection. In each replicate, we randomly selected the same first variable for V_Random_ and V_RandomLowCor_. We selected up to four variables for V_RandomLowCor_, since four was the maximum number of variables that could be selected under the correlation threshold for most North American and Australian species.

### 2.4 Quantification of collinearity and environmental novelty

We calculated two indices for collinearity, the degree of predictor collinearity and collinearity shift. We calculated Pearson's correlation coefficient between each pair of predictors to assess the strength of their correlations, which is the most common approach in quantifying predictor correlation. To calculate the overall degree of collinearity across all predictors used in a model, we first obtained the correlation matrix of predictors in the training region (based on randomly selected background points used in model training; see Section 2.2) and calculated the mean of the absolute values of the upper panel of correlation matrices. We quantified collinearity shift of predictors by calculating the mean absolute differences between the upper panels of the correlation matrices of training and testing regions (see Feng et al., 2015).

Previous studies have shown that model extrapolation in novel environmental conditions can lead to decreased performance (Fitzpatrick et al., 2018; Owens et al., 2013; Qiao et al., 2019). Therefore, we quantified environmental novelty, in essence environmental distance, between testing and training data. More specifically, we first rescaled each climatic variable for each continent (North America and Australia) separately to span one standard deviation across a mean of zero, and then calculated the Euclidean distance between the environmental conditions of background points from training and testing datasets for each modeling replicate. We calculated the distance in two ways: either the mean pairwise distance between background points in training and testing datasets or the mean distance between testing background points and the centroid of training background points. The two measurements of distance were highly correlated (|*r*| = .96), and thus, we only present presented results associated with the latter. Our method of quantifying environmental novelty is comparable to the method of calculating environmental similarity in mobility‐oriented parity (MOP; Owens et al., 2013). Our calculation of distance between training and testing regions corresponds to the similarity between species’ accessible area (M) and projection region (G) in Owens et al. (2013), with the exception that the true extent of species’ accessible area is unknown. Our calculation can be considered as one scenario of using MOP, that is, considering all points in M rather than using a portion of points.

### 2.5 Relationships between model transfer, variable selection, collinearity, environmental novelty, and model performance

We used linear mixed models (lme4 package version 1.1–15 in R; Bates, Mächler, Bolker, & Walker, 2015) to accomplish the three aims. First, we assessed the effects of model transfer scenario (nontransfer vs. transfer) and variable selection (V_Random_ vs. V_RandomLowCor_) on model performance (i.e., AUC, TSS, and sensitivity), using model performance as the dependent variable, transfer scenario and variable selection scheme as fixed effects, and continent and species as nested random effects (Table 1). Similarly, for the second aim, assessing the effects of model transfer scenario on collinearity shift and environmental novelty, and the effects of variable selection on the degree of predictor collinearity, we used the degree of predictor collinearity, environmental novelty, or collinearity shift as dependent variable, model transfer scenario or variable selection as fixed effects, and continent and species as nested random effects. Lastly, to investigate the role of degree of predictor collinearity, collinearity shift, and environmental novelty on model performance, we treated model performance as the dependent variable, degree of predictor collinearity, collinearity shift, and environmental novelty as fixed effects, and continent and species as nested random effects. The dependent or independent variables, if continuous, were rescaled to span one standard deviation around a mean of zero for easier comparison of estimated coefficients (Gelman & Hill, 2007). Though the expected sample size for linear mixed models was 4,960 (31 species * 2 transfer scenarios * 2 variable selection strategies * 10 replicates * 4 folds cross‐validations), the actual sample size was 4,928 because a few modeling replicates failed to meet the variable selection criteria in Section 2.3.

**Table 1:** Summary statistics of linear mixed models. Each row represents a different model, with dependent variables listed on the left and predictors (fixed effects) on the right

Dependent variable	Predictors					
	Intercept	Variable selection (V_Random_ vs. V_RandomLowCor_)	Transfer scenario (Nontransfer vs. Transfer)	Environmental novelty	Degree of predictor collinearity	Collinearity shift
Environmental novelty	−0.38		**0.43** ***			
Degree of predictor collinearity	**0.49** **	**−0.85** ***				
Collinearity shift	**−0.84** ***		**1.69** ***			
TSS	**0.19** ***	0.00	**−0.07** ***			
AUC	**0.69** ***	0.00	**−0.05** ***			
Sensitivity	**0.90** ***	0.00	**−0.08** ***			
TSS	**0.15** ***			**−0.03** ***	0.00	**−0.02** ***
AUC	**0.66** ***			**−0.01** ***	0.00	**−0.01** ***
Sensitivity	**0.85** ***			**−0.03** ***	0.00	**−0.03** ***

Note

Coefficients of covariates are bolded when significant; two decimal places are kept.

*** *p* < .001;

** *p* < .01;

* *p* < .05.

## 3 RESULTS

**Figure 3:**
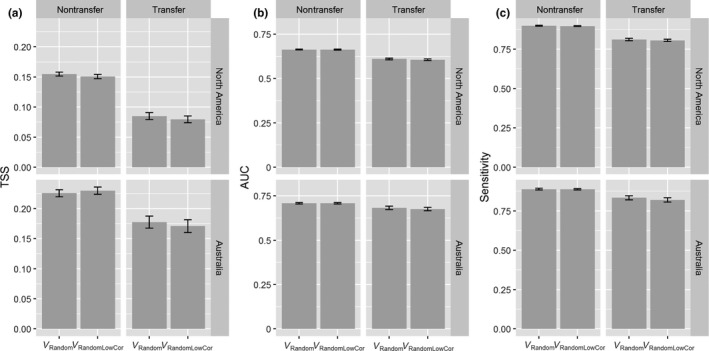
Model performance in transfer and nontransfer scenarios. The scenarios are defined by separating occurrences randomly (nontransfer) or spatially (transfer). Model performance is represented by TSS (panel a), AUC (panel b), and sensitivity (panel c). The data are grouped by study area (North America and Australia) and variable selection strategy (V_Random_ vs. V_RandomLowCor_). Bars represent 95% confidence intervals on the means of models grouped by continent, transfer scenario, and variable selection strategy

The number of spatially unique presences used in ecological niche models ranged from 16 to 922 (mean = 328; median = 260.5) for the 22 North America species and from 21 to 191 (mean = 101.2; median = 94) for the nine Australian species included in this study (Table S2). The performance metrics (AUC, TSS, and sensitivity) indicated that the models based on the random data partition into training and testing (nontransfer scenario) performed well, on average, for all species studied (Figure 3).

**Figure 4:**
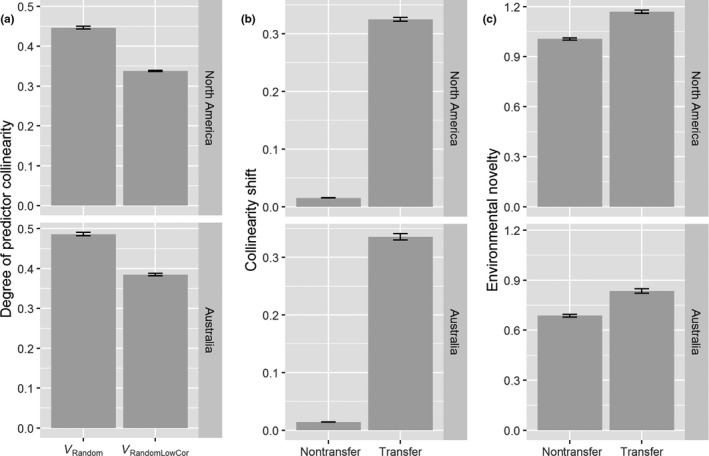
Summary of degree of predictor collinearity (a) in different variable selection strategy (V_Random_ vs. V_RandomLowCor_) and collinearity shifts (b) and environmental novelty (c) under model transfer versus nontransfer scenarios. Bars represent 95% confidence intervals

**Figure 5:**
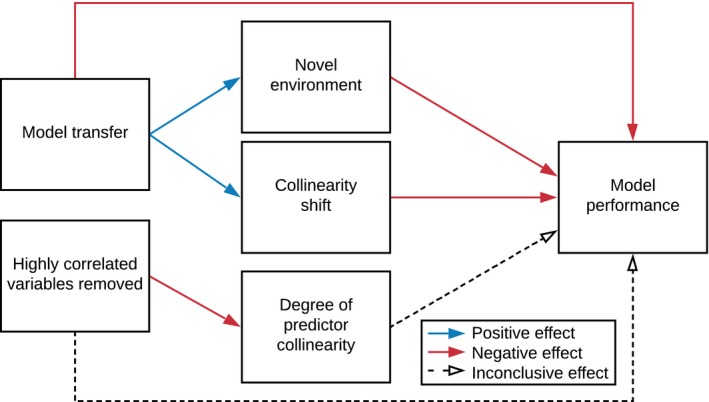
Conceptual summary of results. Solid lines represent significant relationships (blue for positive effects and red for negative effects) supported by our results; dashed lines represent inconclusive relationships

We found that transferring models led to significantly lower model performance, indicated by lower AUC, TSS, and sensitivity values compared with the nontransfer scenario (Table 1). Model transfer also led to significantly higher collinearity shifts and higher environmental novelty (Figure 4). Excluding highly correlated variables led to significantly lower degree of predictor collinearity (Figures 4 and 5; Table 1), but had little effect on model performance in both nontransfer and transfer scenarios (Figures 3 and 5; Table 1).

The linear mixed models showed that both degree of collinearity shift and environmental novelty had a negative effect on model performance, while predictor collinearity was not correlated with model performance (Table 1).

## 4 DISCUSSION

Despite frequent mentions in the literature, the effects of predictor collinearity on Maxent models have not been well understood; thus, approaches toward documenting and dealing with collinearity have been arbitrary. Our study clarifies whether, when, and how collinearity affects model performance in Maxent. First, we show decreased model performance in model transfer scenarios, a well‐known phenomenon observed in many studies (Fitzpatrick et al., 2018; Owens et al., 2013; Qiao et al., 2019). The potential underlying mechanisms are likely the degree of predictor collinearity, collinearity shift, and environmental novelty. To clarify the role of these potential mechanisms, we further showed that model transfer was accompanied by considerably increased collinearity shift and environmental novelty, both of which were associated with decreased model performance. The degree of predictor collinearity can be controlled by removing highly correlated variables, but in our study this approach did not affect model performance, providing direct evidence of Maxent's ability to regulate model complexity by downplaying the importance of redundant variables. This finding is also confirmed by De Marco Júnior and Nóbrega (2018) using simulated data. However, collinearity shift and environmental novelty are expected to be independent of variable selection strategy and dependent on the environmental difference between the training and projecting regions. Therefore, even though Maxent can regulate the contribution of redundant variables, it is not immune to collinearity shift and environmental novelty, which is independent of the modeling algorithm and can lead to lower predictive performance when models are transferred. In other words, the strategy of removing highly correlated variables does not help improve Maxent models, because (a) Maxent is able to regulate redundant variables and alleviate the effects of variable collinearity on model training, and (b) collinearity shift and environmental novelty are independent of the degree of predictor collinearity.

### 4.1 The degree of predictor collinearity versus collinearity shift

It is important to distinguish between the roles of degree of predictor collinearity and collinearity shift. The former may impact model estimation, whereas the latter impacts the accuracy of model prediction in the testing region. Both aspects can negatively impact the accuracy of classical regression models, but Maxent can balance the trade‐off between model fit and model complexity through regularization (Elith et al., 2011); therefore, the degree of predictor collinearity is not expected to affect Maxent.

Collinearity shift can occur when training and testing data are environmentally different. In the context of ENM, models are frequently transferred to different regions and/or time points, so collinearity shifts are likely common in ENM applications. The magnitude of collinearity shift depends on the difference between training and testing data. But can collinearity shifts be reduced by removing highly correlated variables in model training? Probably not, because one could not predict the change in correlation between a pair of predictors, since two highly correlated variables will not necessarily experience more correlation shift than a pair of less correlated variables. From another perspective, the collinearity shift of a predictor set will always be predetermined when the training and testing data are decided in the stage of experimental design, before model training and projecting.

### 4.2 Collinearity in Maxent modeling

Our results supported the view that Maxent is robust to the degree of predictor collinearity (Elith et al., 2011) in the context of model training. However, given the role of collinearity shift and the independence between degree of predictor collinearity and collinearity shift, Maxent is not totally immune to issues of collinearity. Our results showed that removing highly correlated variables did not significantly influence the accuracy of Maxent model (Table 1), regardless of model transfer scenario, because Maxent can regulate the contribution of redundant predictors; the aspect that matters more in Maxent modeling is the collinearity shift in model transfer scenarios; therefore, we recommend to quantify the collinearity shift as a proxy of model accuracy (e.g., Feng et al., 2015).

While the effects of collinearity are well understood in classical regression models (Dormann et al., 2013), they remain inconclusive in even the most recent Maxent publications (Appendix S1). We think that the different roles of degree of predictor collinearity and collinearity shift, model transfer scenario, and difference in parameter estimation between Maxent and classical regression models may all have contributed to the confusion of collinearity in the Maxent modeling community.

### 4.3 Model transfer is challenging

Model transfer is essentially challenging and even risky (Gelman & Hill, 2007), as evidenced by the dramatically decreased model performance when our Maxent models were projected to different regions. Previous studies on ENM transferability mainly examined the negative impact of novel environments on model performance, as the estimated relationship between species distribution and environmental predictors may be invalid in other, nontraining environments (Fitzpatrick et al., 2018; Owens et al., 2013; Qiao et al., 2019). Here, we also find a negative impact of collinearity shift on model performance in transfer scenarios, as the relationships between predictors in the training area do not necessarily apply in the projected area.

### 4.4 Future research

In our study design, we selected variables based on the correlation of predictors to mimic a common practice in ENM literature (De Marco Júnior & Nóbrega, 2018). However, generally speaking, the approach of selecting less correlated predictors does not fully solve the collinearity issue as even a low level of collinearity can bias the ecological models (Graham, 2003). Moreover, this approach faces two issues: the chance of ignoring the unique contributions of omitted variables and the inferential problem in deciding which variable to drop between a highly correlated pair (Graham, 2003). Alternative approaches have been proposed to solve the issue through the functional nature of collinearity. For example, the principal component analysis (PCA) assumes shared contributions from correlated predictors and extracts vectors to account for the variations of predictors, but the major limitation of PCA is the lack of biological interpretation of the principal components (Graham, 2003). Besides the limitation of interpretability, the PCA approach, when used for future predictions, still suffers from the issue of collinearity shift during model transfer. This is due to the fact that the principal components are determined by the eigen‐structure of the sample covariance matrix of the predictors (Abdi & Williams, 2010), and the collinearity shift will distort the original eigen‐structure and hence change the principal components in a different spatial and temporal context.

Though we focused on Maxent in our study with the aim to capture a common practice in ENM literature, many other algorithms are used in ENM literature (e.g., 33; Norberg et al., 2019). The vulnerability to degree of predictor collinearity should vary with and depend on the mechanisms in each algorithm. According to comparisons done by De Marco Júnior and Nóbrega (2018), envelope algorithms are more sensitive to degree of collinearity, compared with more complex algorithms, such as Maxent. Comprehensive comparisons and evaluations of sensitivity of algorithms to collinearity are still rare in general and thus require more investigation. Nevertheless, the negative effects of collinearity shift and novel environments are likely generalizable to other modeling algorithms, because those issues are dependent on the choice of training and projection data, and independent of modeling algorithms.

Our experimental design reflects common practices used in Maxent modeling (e.g., variable selection based on correlation coefficients, default Maxent parameters, and widely used climatic dataset); thus, the results have broad implications for Maxent applications. Also, our study was conducted across two continents with varied climatic regimes. The use of real landscapes makes our study more likely to capture the complexities that are commonplace in empirical studies. It is worth reflecting on how frequently collinearity shift and novel environments are coupled or decoupled. In our study, the scenario of model transfer was the major driver of collinearity shift and novel environments, suggesting that the presence of collinearity shift and novel environments could be commonly coupled during the model transfer (Figure 4). This is probably true in general simply because of the heterogeneous landscape on Earth, that is, different areas rarely have the same environments. However, in the transfer scenario, the strength of collinearity shift and environmental novelty showed very weak correlation in our case (Figure S1), suggesting the magnitude of both is likely decoupled. In other words, the magnitude of change in correlation of a pair of highly correlated variables should depend on the modeling context, defined by predictor selection and spatial and temporal extent and resolution of the environmental predictors (Jiménez‐Valverde, Nakazawa, et al., 2009).

In contrast with using data from real world, there is an increasing trend of using virtual species and even virtual landscapes in methodological explorations in ENM (Feng & Papeş, 2017; Hirzel, Helfer, & Metral, 2001; Leroy, Meynard, Bellard, & Courchamp, 2016; Meynard & Kaplan, 2013; Moudrý, 2015; Qiao et al., 2016). Notably, De Marco Júnior and Nóbrega (2018) studied the influence of degree of predictor collinearity using virtual species that have defined niches, with the obvious advantage of knowing the species’ true distribution in model evaluation. Their study reached a similar conclusion on the robustness of Maxent on collinearity; in addition, the study had an expanded scope on multiple modeling algorithms and found different levels of algorithm sensitivity to the issue of collinearity. Similarly, future research could validate our findings using virtual species or using a simulated landscape with well‐controlled environmental conditions, and examine the role of collinearity shift and novel environments on ENM algorithms beyond Maxent, as well as explore different approaches in handling collinearity. Nonetheless, by basing our investigations on empirical data, we highlight the issues that are likely to be present in studies dealing with real‐world systems.

## 5 CONCLUSIONS

Based on our analyses, we draw the following three conclusions: (a) Maxent is capable of regulating contributions from redundant variables, rendering its robustness to degree of predictor collinearity in model training; (b) Maxent is not immune to environmental novelty and collinearity shifts, and we thus recommend estimation of these factors to better infer uncertainties when models are spatially and/or temporally transferred; and (c) the strategy of removing highly correlated variables has little impact in Maxent model performance because of the way Maxent deals with redundant variables and the independence between degree of predictor collinearity and collinearity shift.

## CONFLICT OF INTEREST

None declared.

## AUTHORS’ CONTRIBUTIONS

All authors conceived the study; X.F. and R.P. prepared the data; X.F. conducted the analyses with help from D.S.P. and Y.L.; X.F. drafted the manuscript; and all authors interpreted the results and contributed to the revision of the manuscript.

## Data Availability

Details of Google Scholar literature search and summary of mammal species are in Supporting Information. The occurrences and climatic data are available from https://doi.org/10.5281/zenodo.3352333.
