# Supplementary material for: Collinearity in ecological niche modeling: Confusions and challenges
Source: Ecol Evol. 2019 Aug 20;9(18):10365–76. doi: 10.1002/ece3.5555 (PMC6787792; doi:10.1002/ece3.5555)
Supplement: Supplementary file 1 [file ECE3-9-10365-s001.docx]

**Appendix S1.** Details of Google Scholar literature search for papers published in 2017 that have cited three major Maxent publications (Phillips et al., 2004; Phillips et al., 2006; Phillips & Dudík, 2008).

We identified three major Maxent papers that have been collectively cited ~12,000 times (Google Scholar, accessed 6 November, 2017) and focused on publications that have cited any of the three Maxent papers. To represent the current status of knowledge, we restricted our search to journal articles published in 2017. We also restricted our focus to literature published in English. For each Maxent paper, we first recorded the total number of citations in 2017. To identify publications that are relevant to Maxent and collinearity, we further filtered the citations of each Maxent publication and recorded the number of publications that met the following searching criteria via Google Scholar: “collinearity” and/or “variable correlation”. After identifying the number of citations for each Maxent publication, we calculated the total number of unique citations for each search criterion (i.e., a publication is only considered once if it cites two or more Maxent publications).

**Figure S1.** Scatter plot of collinearity shift and environmental novelty in different transfer scenario and continent. The blue line represents the linear relationship, with the shaded area represents 95% confidence interval.


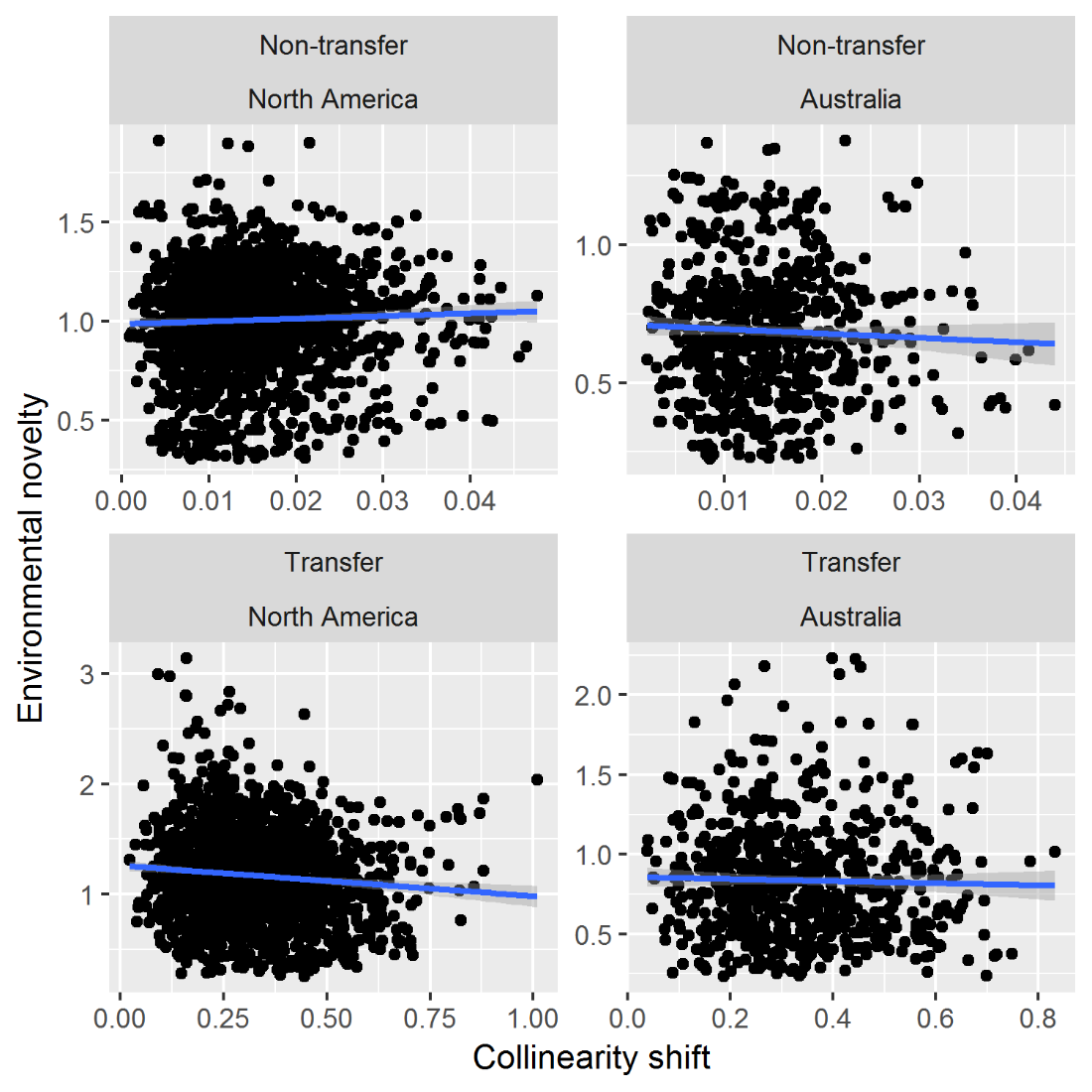


**Table S1.** Summary of Google Scholar literature search of papers published in 2017 that have cited at least one of the three main Maxent papers. “Total” represents number of unique publications (i.e., a publication is only considered once if it cites two or more Maxent publications).

| Maxent publications | Number of papers citing Maxent publications | Number of papers mentioning “collinearity” | Number of papers mentioning “variable correlation” | Number of papers mentioning “collinearity” and “variable correlation” | Number of papers mentioning “collinearity” or “variable correlation” |
| --- | --- | --- | --- | --- | --- |
| [Phillips et al. (2004)](#_ENREF_42) | 125 | 24 | 1 | 0 | 25 |
| [Phillips et al. (2006)](#_ENREF_39) | 847 | 177 | 5 | 2 | 180 |
| [Phillips and Dudík (2008)](#_ENREF_40) | 347 | 81 | 2 | 0 | 83 |
| Total | 980 | 202 | 5 | 2 | 205 |

**Table S2.** Summary of mammal species included in this study, with number of occurrences used in ecological niche models.

| **Order** | **Family** | **Scientific name** | **Spatially**  **unique**  **occurrences** | **Geographic area** |
| --- | --- | --- | --- | --- |
| Carnivora | Canidae | *Canis latrans* Say, 1823 | 922 | North America |
| Carnivora | Canidae | *Urocyon cinereoargenteus* (Schreber, 1775) | 568 | North America |
| Carnivora | Canidae | *Vulpes macrotis* Merriam, 1888 | 98 | North America |
| Carnivora | Canidae | *Vulpes velox* (Say, 1823) | 58 | North America |
| Carnivora | Felidae | *Lynx canadensis* Kerr, 1792 | 240 | North America |
| Carnivora | Felidae | *Lynx rufus* (Schreber, 1777) | 667 | North America |
| Carnivora | Mephitidae | *Conepatus leuconotus* (Lichtenstein, 1832) | 138 | North America |
| Carnivora | Mephitidae | *Mephitis macroura* Lichtenstein, 1832 | 93 | North America |
| Carnivora | Mephitidae | *Mephitis mephitis* (Schreber, 1776) | 571 | North America |
| Carnivora | Mephitidae | *Spilogale gracilis* Merriam, 1890 | 82 | North America |
| Carnivora | Mephitidae | *Spilogale putorius* (Linnaeus, 1758) | 112 | North America |
| Carnivora | Mustelidae | *Lontra canadensis* (Schreber, 1777) | 291 | North America |
| Carnivora | Mustelidae | *Martes americana* (Turton, 1806) | 281 | North America |
| Carnivora | Mustelidae | *Martes pennanti* (Erxleben, 1777) | 106 | North America |
| Carnivora | Mustelidae | *Mustela frenata* Lichtenstein, 1831 | 627 | North America |
| Carnivora | Mustelidae | *Mustela nigripes* (Audubon & Bachman, 1851) | 16 | North America |
| Carnivora | Mustelidae | *Neovison vison* (Schreber, 1777) | 523 | North America |
| Carnivora | Mustelidae | *Taxidea taxus* (Schreber, 1777) | 409 | North America |
| Carnivora | Procyonidae | *Bassariscus astutus* (Lichtenstein, 1830) | 202 | North America |
| Carnivora | Procyonidae | *Nasua narica* (Linnaeus, 1766) | 86 | North America |
| Carnivora | Procyonidae | *Procyon lotor* (Linnaeus, 1758) | 768 | North America |
| Carnivora | Ursidae | *Ursus americanus* Pallas, 1780 | 370 | North America |
| Diprotodontia | Macropodidae | *Macropus agilis* (Gould, 1842) | 62 | Australia |
| Diprotodontia | Macropodidae | *Macropus antilopinus* (Gould, 1842) | 31 | Australia |
| Diprotodontia | Macropodidae | *Macropus dorsalis* (Gray, 1837) | 41 | Australia |
| Diprotodontia | Macropodidae | *Macropus fuliginosus* (Desmarest, 1817) | 155 | Australia |
| Diprotodontia | Macropodidae | *Macropus giganteus* Shaw, 1790 | 176 | Australia |
| Diprotodontia | Macropodidae | *Macropus parryi* Bennett, 1835 | 21 | Australia |
| Diprotodontia | Macropodidae | *Macropus robustus* Gould, 1841 | 191 | Australia |
| Diprotodontia | Macropodidae | *Macropus rufogriseus* (Desmarest, 1817) | 94 | Australia |
| Diprotodontia | Macropodidae | *Macropus rufus* (Desmarest, 1822) | 140 | Australia |
